# Cross-Reactive Antibodies in Tick-Borne Encephalitis: Case Report and Literature Review

**DOI:** 10.3390/antib11040072

**Published:** 2022-11-20

**Authors:** Tatjana Vilibic-Cavlek, Thomas Ferenc, Mateja Vujica Ferenc, Maja Bogdanic, Tanja Potocnik-Hunjadi, Dario Sabadi, Vladimir Savic, Ljubo Barbic, Vladimir Stevanovic, Federica Monaco, Eddy Listes, Giovanni Savini

**Affiliations:** 1 Department of Virology, Croatian Institute of Public Health, 10000 Zagreb, Croatia; 2 School of Medicine, University of Zagreb, 10000 Zagreb, Croatia; 3 Clinical Department of Diagnostic and Interventional Radiology, Merkur University Hospital, 10000 Zagreb, Croatia; 4 Institute of Emergency Medicine of Krapina-Zagorje County, 49000 Krapina, Croatia; 5 Department for Infectious Diseases, General Hospital Varazdin, 42000 Varazdin, Croatia; 6 Department for Infectious Diseases, Clinical Hospital Center Osijek, 31000 Osijek, Croatia; 7 Medical Faculty, Josip Juraj Strossmayer University of Osijek, 31000 Osijek, Croatia; 8 Laboratory for Virology and Serology, Poultry Center, Croatian Veterinary Institute, 10000 Zagreb, Croatia; 9 Department of Microbiology and Infectious Diseases with Clinic, Faculty of Veterinary Medicine, University of Zagreb, 10000 Zagreb, Croatia; 10 Department of Virology, OIE Reference Center for West Nile Disease, Istituto Zooprofilattico Sperimentale “G. Caporale”, 64100 Teramo, Italy; 11 Laboratory for Diagnostics, Croatian Veterinary Institute, Veterinary Institute Split, 21000 Split, Croatia

**Keywords:** tick-borne encephalitis virus, West Nile virus, Usutu virus, flaviviruses, cross-reactive antibodies

## Abstract

Flaviviruses are a heterogeneous group of viruses that may induce broad antigenic cross-reactivity. We present a patient who was admitted to the infectious disease department with symptoms suggestive of aseptic meningitis. During the clinical workup, the patient reported a tick bite two weeks before the disease onset. High titers of IgM and IgG antibodies to tick-borne encephalitis virus (TBEV) were found in both serum and cerebrospinal fluid (CSF) samples, indicating acute TBEV infection. West Nile virus (WNV) and Usutu virus (USUV) IgM and/or IgG antibodies were also detected, and a virus neutralization test (VNT) was performed. A high titer of TBEV neutralizing (NT) antibodies (640) was detected, which confirmed acute TBE. However, NT antibodies to WNV and USUV were also detected (titer 80 for both viruses). After TBEV and WNV IgG avidity evaluation, previous flavivirus infection was highly suspected (avidity index 82% and 89%, respectively). Blood, CSF, and urine samples were negative for respective viruses’ RNA. The presented case highlights the challenges in flavivirus serodiagnosis. In the published literature, different degrees of cross-reactivity or cross-neutralization between TBEV and dengue, louping ill, Omsk hemorrhagic fever, Langat, and Powassan virus were also observed. Therefore, the serology results should be interpreted with caution, including the possibility of cross-reactivity. In areas where several flaviviruses co-circulate VNT is recommended for disease confirmation.

## 1. Introduction

Tick-borne encephalitis (TBE) is a neuroinvasive infection caused by the tick-borne encephalitis virus (TBEV), which belongs to the family *Flaviviridae*, genus *Flavivirus*. The genus *Flavivirus* is composed of more than 70 viral species, organized into serocomplexes. TBEV belongs to the tick-borne encephalitis serocomplex [1]. There are three subtypes of TBEV: European, Far Eastern, and Siberian. The disease is endemic in wide areas of Europe and Northeastern Asia. Nowadays, TBE is one of the most important re-emerging zoonoses in Europe [2]. Human TBE infections mostly occur after the bite of infected *Ixodes* ticks; however, there are an increasing number of cases or case series of patients infected after consumption of raw milk from infected goats, sheep, or cows [3,4]. Infections caused by European TBEV typically manifest as a biphasic disease. The first phase is characterized by fever and non-specific symptoms, while in the second phase neurological symptoms might occur due to the involvement of the central nervous system (CNS) and this is usually the time when the patients are hospitalized [5]. Since TBEV RNA only rarely can be detected in serum or cerebrospinal fluid (CSF) at the time of onset of the neurological phase, diagnosis of TBEV is most commonly confirmed by serology, usually enzyme-linked immunosorbent assay (ELISA) [2]. In almost all patients, IgM and often IgG can be detected at the beginning of neurological symptoms [6]. However, in some patients, TBEV IgM antibodies may persist for several months after infection [5]. In addition, due to antigenic similarity, IgM antibodies may be cross-reactive, induced by other flaviviruses, especially among viruses within the same serocomplex. Therefore, an early diagnosis of TBEV confirmed by detecting IgM alone is sometimes questionable. Cross-reactive flavivirus IgG antibodies are more frequently detected, which may lead to a misinterpretation of the serological results in the case of another ongoing flavivirus CNS disease [5]. 

Although a virus neutralization test (VNT) is a more specific serology test, some degree of cross-reactions with other flaviviruses either neutralizing or enhancing the infection was also observed [7]. Cross-reactions in VNT are more common among West Nile virus (WNV) and Usutu virus (USUV), since these viruses are antigenically closer by genomic phylogeny compared to TBEV [8]; however, cross-reactivity with TBEV has also been observed [9].

We present a case of TBE detected in a patient with a previous flavivirus infection to point out diagnostic difficulties in flavivirus serodiagnosis. In addition, a literature review on flavivirus cross-reactivity was performed in PubMed/MEDLINE using the combination of keywords “Cross-reactive antibodies” and “Tick-borne encephalitis virus”. There were no restrictions regarding language or publishing year. A list of original articles, reviews, and case reports was assembled and studies appearing to meet the inclusion criteria were reviewed in full text. Additional studies were found by reviewing reference lists of retrieved research papers. 

## 2. Case Report

In August 2019, a male patient in his early twenties was admitted to the infectious disease department with symptoms suggestive of aseptic meningitis (fever up to 39 °C, headache, and vomiting). The patient resided in a rural area of North-West Croatia with documented flavivirus circulation (TBEV, WNV, and USUV) [10,11]. The patient reported no history of recent travel or vaccination against TBE but reported a tick bite two weeks before the disease onset. Routine laboratory parameters were within the normal range (Table 1). CSF analysis showed pleocytosis (280 leukocytes, 80% mononuclear cells), elevated protein level (0.637 g/L), and normal glucose level (3.0 mmol/L). 

Serum, blood, CSF, and urine samples were collected on day 9 after disease onset. Serum and CSF samples were tested for the presence of TBEV, WNV, and USUV IgM and/or IgG antibodies using commercial ELISA kits (Euroimmun, Lübeck, Germany; Focus Diagnostic, Cypress, CA). Additionally, blood, CSF, and urine samples were tested for the presence of viral RNA: TBEV (Schwaiger et al., 2003) [12], WNV (Tang et al., 2006) [13], and USUV (Nikolay et al., 2014) [14].

Serology and virology results are presented in Table 2. High titers of TBEV antibodies were found using ELISA in both serum (IgM ratio 3.62, positive >1.1; IgG 161.90 RU/mL, positive >22) and CSF (IgM ratio 3.65, IgG 146.50 RU/mL) samples, indicating acute TBEV infection. Since WNV and USUV IgM and/or IgG antibodies were also detected, a VNT was performed. The TBEV Ljubljana strain was used as an antigen for VNT. The virus titer (median tissue culture infectious dose; TCID_50_) was calculated on day 5 after inoculation using the Reed and Muench formula. The serum sample was heat-inactivated (30 min/56 °C), and serial two-fold dilutions starting at 1:5 were prepared in duplicate in a 96-well microtiter plate using Dulbecco’s Modified Eagle Medium (DMEM; Lonza, Basel, Switzerland). An equal amount (25 μL) of inactivated serum dilutions and 100 TCID_50_ of TBEV were mixed and incubated for 1 h at 37 °C. In the last phase, 50 μL of 2 × 10^5^ Vero E6 cells/mL in DMEM with 10% of heat-inactivated fetal calf serum (Capricorn Scientific, Ebsdorfergrund, Germany) were added to each well. To ensure optimal testing results, virus suspension (incubated at 37 °C with CO_2_ for 1 h) was back titrated in four tenfold dilutions: 100, 10, 1, and 0.1 TCID_50_. Positive and negative control were included. The plate was incubated at 37 °C with CO_2_ for five days and examined for the cytopathic effect starting from the third day of incubation. Antibody titer was defined as the reciprocal value of the highest dilution of the serum that showed 100% neutralization. Titer ≥ 1:10 was considered positive [15]. In the VNT, a high titer of TBEV (640) was recorded, confirming acute TBE. However, neutralizing titers to WNV (80) and USUV (80) were also detected [16]. The serum sample was further tested for TBEV and WNV IgG avidity (Euroimmun, Lübeck, Germany). Both TBEV and WNV IgG antibodies were of high avidity (82% and 89%, respectively; reference values <40% low avidity, 40–60% borderline avidity, >60% high avidity), which suggested a previous flavivirus infection. Blood, CSF, and urine samples were negative for TBEV, WNV, and USUV RNA.

## 3. Discussion

Flaviviruses are usually divided into three subgroups based on their transmission routes: mosquito-borne (e.g., yellow fever virus (YFV), Japanese encephalitis virus (JEV), Zika virus (ZIKV), dengue virus (DENV), WNV), tick-borne (e.g., Powassan virus (POWV), louping ill virus (LIV), TBEV), and no-known-vector flaviviruses (e.g., Rio Bravo virus) [17,18,19]. Furthermore, flaviviruses are organized into eight serocomplexes based on their cross-creative characteristics which are closely related to genomic phylogeny [17,20]. Two of the largest serocomplexes are tick-borne encephalitis virus complex, which includes TBEV, Omsk hemorrhagic fever virus (OHFV), Langat virus (LGTV), POWV, etc.), and Japanese encephalitis virus complex (WNV, USUV, JEV, etc.), while for instance YFV, DENV, and ZIKV are in separate serocomplexes, respectively [17]. 

Innate and adaptive immune responses, especially induction of early antibody response, are essential to neutralize flavivirus infection. The surface envelope glycoprotein (E-protein) is the main target for the antibody response to flaviviruses which may result in the production of cross-reactive, non-neutralizing antibodies, particularly within the same serocomplex due to the protein structure that contains virus-specific and cross-reactive epitopes [9,17,20]. There are three structural domains of the E-protein, annotated I–III. Many virus-specific neutralizing antibodies aim at the epitopes on domain III (DIII), whereas the fusion loop on the tip of domain II (DII) is responsible for the majority of cross-reactive antibodies [21,22,23]. Domain I (DI) is the central part of the E-protein responsible for the orientational stabilization of the protein, while it is also a carrier of the N-linked glycosylation site that is associated with virus replication, pH sensitivity, and neural invasion [24].

The TBE diagnosis relies on both clinical and laboratory findings. According to the definitions of the European Center for Disease Prevention and Control (ECDC), criteria for a confirmed TBE case include both clinical (symptoms of inflammation of the CNS) and laboratory criteria (TBEV IgM and IgG antibodies in serum, TBEV IgM antibodies in the CSF or detection of TBEV RNA in clinical specimen) [25]. Humans represent dead-end hosts for many flaviviruses, including TBE; therefore, diagnosis is mainly confirmed using serology, as the viremic phase is rather short [26]. 

In the reported case, neurological symptoms (meningitis), as well as the presence of high levels of TBEV IgM and IgG antibodies found in both serum (3.62 and 161.90, respectively) and CSF (3.65 and 146.50, respectively) samples supported the diagnosis of acute TBEV infection which was further confirmed by the high titer of neutralizing antibodies obtained using a VNT (640), a more specific serology test. The possible co-circulation of several flaviviruses in the same area represents a diagnostic challenge due to the broad antibody cross-reactivity within and between the different serocomplexes [7]. 

The cross-reactivity of TBEV with other flaviviruses is presented in Table 3. An earlier study by Allwinn et al. [27] (2002) found that 9.5% of TBEV-vaccinated individuals showed cross-reactive IgG antibodies to DENV. High cross-reactivity with DENV was also noted in patients with acute TBEV infection. Strong IgM cross-reactivity could also be seen between WNV and DENV, and to a lesser degree between WNV and TBEV due to their transmission and serocomplex distinction [9]. In one German study, field serum samples or vaccinated serum samples from animals positive for various flaviviruses were tested for cross-reactivity patterns (sheep, equines, ducks, chickens, rabbits, and mice). The results showed cross-reactivity between LIV and TBEV, as well as between TBEV and WNV [3]. Maeki et al. [18] demonstrated that serum samples from JEV-positive patients displayed cross-reactive antibodies to WNV, DENV, and TBEV in IgM and/or IgG ELISA. However, only WNV showed some degree of cross-reactivity to JEV in the neutralization tests which emphasizes their importance for diagnosis confirmation of flavivirus infections. A recent study by Stiasny et al. [28] detected higher titers of cross-reactive antibodies after DENV infection in comparison to ZIKV and TBEV infections. A possible explanation for this phenomenon is the greater exposure of the fusion loop of domain II in DENV [22,28].

Therefore, previous exposure to related flaviviruses may interfere with the serology analysis resulting in false positive test results. In addition to TBEV antibodies, IgM/IgG antibodies to WNV and IgG antibodies to USUV were also detected in the patient presented in this report in both ELISA and VNT. Regarding the WNV/USUV positive antibodies, there are several possible scenarios: (I) no WNV/USUV previous infections, WNV and USUV antibodies were results of cross-reactions: weak positive WNV IgM (1.24), similar neutralizing titers of WNV (80) and USUV (80); (II) previous quite recent asymptomatic WNV infection: weak positive WNV IgM (1.24), relatively high VNT titer (80), high IgG avidity (89%), the USUV antibodies were due to cross-reaction to TBEV/WNV; (III) possible USUV previous infection: relatively high titer in VNT (80), WNV antibodies were due to cross-reactions; (IV) possible quite recent WNV infection and previous USUV infection: weak positive WNV IgM (1.24), relatively high titers in VNT (WNV 80, USUV 80). Although there is a possibility that TBEV-neutralizing antibodies cross-neutralized WNV and USUV, the detection of high-avidity TBEV and WNV IgG antibodies supported a probable previous flavivirus exposure in the patient presented in this study.

Over the years, the increase in vaccination and use of monoclonal antibodies against flavivirus infections has inspired researchers to investigate cross-reactivity and possible simultaneous protective effects against several other members of this virus family. Mansfield et al. [20] found that patients who had been immunized against YFV, JEV, and TBEV had a greater likelihood of neutralizing WNV compared to those who had only been immunized against JEV and TBEV. Two groups of authors have demonstrated that the TBEV vaccine may also provide efficient protection against OHFV infection in humans [22,29]. Conversely, research by Malafa et al. [23] showed that prior vaccine-induced flavivirus immunity (against YFV and TBEV) resulted in higher levels of cross-reactive antibodies but lower levels of specific-IgM antibodies in primary ZIKV infection. Other authors have reported that prior YFV vaccination increased titers of cross-reactive antibodies but impaired the production of specific neutralizing antibodies in the TBE vaccination group [30]. Overall, all the study participants had adequate post-vaccination neutralizing titers. A study by Agudelo et al. [19] reported that hospitalized individuals with natural TBEV infection demonstrated substantial binding of neutralizing antibodies to EDIII and more potent neutralizing activity compared to the vaccinated cohort. These natural antibodies neutralized several flaviviruses within the same serocomplex. Studies have shown that cross-reactive immunity may also be beneficial in the JEV serocomplex [17]. A recent study by VanBlargan et al. [31] developed monoclonal antibodies against POWV that showed cross-reactivity and an in vivo protective effect against serocomplex similar flaviviruses (TBEV, LGTV).

## 4. Conclusions

In conclusion, the presented case indicates that serologic diagnosis of flaviviruses can be challenging. ELISA and VNT antibody test results can sometimes be difficult to interpret, particularly in secondary flavivirus infections. A previous flavivirus infection in the patient presented in this report affected the antibody response in the current TBEV infection (high avidity IgG antibodies). Since patients do not always notice tick bites, the serodiagnosis could have been directed solely to WNV, particularly in individuals who reside in areas with documented WNV circulation and exhibited symptoms at the peak of WNV transmission activity. Therefore, the results of flavivirus serology should be carefully interpreted, including analysis of possible cross-reactivity. In addition, the presented results emphasize the need for confirmatory testing by VNT, especially in patients from areas where several flaviviruses co-circulate.

## Figures and Tables

**Table 1:** Laboratory results of patient samples.

Sample	Parameter	Value	Reference Range
Blood	Red blood cells (×10^12^/L)	4.67	4.4–5.8
	Hemoglobin (g/L)	133	120–180
	C-reactive protein (mg/L)	29.4	<5.0
	While blood cells (×10^9^/L)	7.2	4.0–10.0
	Platelets (×10^9^/L)	331	100–400
	Aspartate-aminotransferase (U/L)	20	11–38
	Alanine-aminotransferase (U/L)	20	12–48
	Gamma-glutamyl transferase (U/L)	14	11–55
Cerebrospinal fluid	Cell count/mm^3^	280	<5
	Polymorphonuclear cells (%)	20	
	Mononuclear cells (%)	80	100%
	Proteins (g/L)	0.637	0.170–0.370
	Glucose (mmol/L)	3.0	2.5–3.3

**Table 2:** Flavivirus serology and virology results of patient samples.

Virus	Serology				RT-PCR		
	Serum ELISA IgM ^a^/IgG ^b^	IgG Avidity	CSF ELISA IgM ^a^/IgG ^b^	Serum VNT ^c^	Blood	CSF	Urine
TBEV	Positive (3.62)/Positive (161.90)	82%	Positive (3.65)/Positive (146.50)	640	Negative	Negative	Negative
WNV	Positive (1.24)/Positive (193.44)	89%	Negative (0.23)/Positive (139.86)	80	Negative	Negative	Negative
USUV	NT/Positive (>200)	NT	NT/Positive (167.25)	80	Negative	Negative	Negative

TBEV = Tick-borne encephalitis virus; WNV = West Nile virus; USUV = Usutu virus; NT = not tested; ^a^ ratio < 0.8 negative, 0.8–1.1 borderline, >1.1 positive; ^b^ RU/mL <16 negative, 16–22 borderline, >22 positive; ^c^ titer.

**Table 3:** Cross-reactivity of TBEV with other members of the *Flaviviridae* family.

Country	Flavivirus	% Cross-Reactivity	Method	Reference
Germany	DENV (1–4)	IgG 25.9%—TBE patients IgG 9.5%—vaccinated individuals	ELISA	Allwinn et al. (2002) [27]
United Kingdom	LIV	68%		Mansfield et al. (2011) [20]
	DENV-2	38.5%	VNT	
	WNV	7.1%—vaccinated against TBEV and JEV, 32.1%—vaccinated against TBEV, JEV, and YFV		
Austria	OHFV	98–100%	VNT	Orlinger et al. (2011) [29]
Japan	OHFV	79–86%	VNT	Chidumayo et al. (2014) [22]
Germany	LIV	80% ^1^	ELISA/VNT	Klaus et al. (2014) [3]
	WNV	26% ^1^		
Croatia	WNV USUV	IgG 15.4%; IgM 8.0% IgG 13.5%	ELISA	Project CRONEUROARBO (2017–2021), unpublished data
United States of America, United Kingdom, Czech Republic	LGTV, LIV, OHFV, POWV, KFDV	Neutralizing activity detected	VNT	Agudelo et al. (2021) [19]

^1^ Animal samples (sheep, horses, respectively). ELISA = enzyme-linked immunosorbent assay; VNT = virus neutralization test; DENV = dengue virus, LIV = louping ill virus, WNV = West Nile virus, TBEV = tick-borne encephalitis virus; JEV = Japanese encephalitis virus; OHFV = Omsk hemorrhagic fever, USUV = Usutu virus; LGTV = Langat virus, POWV = Powassan virus, KFDV = Kyasanur Forest disease virus, YFV = yellow fever virus.

## Data Availability

Data sharing not applicable.
